# Supplementary material for: Bioinspired porous three-coordinated single-atom Fe nanozyme with oxidase-like activity for tumor visual identification via glutathione
Source: Nat Commun. 2023 Nov 6;14:7127. doi: 10.1038/s41467-023-42889-w (PMC10638392; doi:10.1038/s41467-023-42889-w)
Supplement: Supplementary file 1 — Supplementary Information [file 41467_2023_42889_MOESM1_ESM.pdf]

Supplementary Information

Bioinspired porous three-coordinated single-atom Fe nanozyme with oxidase-like activity for tumor visual identification *via* glutathione

Da Chen[1], Zhaoming Xia[2], Zhixiong Guo[1], Wangyan Gou[1], Junlong Zhao[3], Xuemei Zhou[4], Xiaohe Tan[1], Wenbin Li[1], Shoujie Zhao[3], Zhimin Tian[1,*] and Yongquan Qu[1,*]

[1]Key Laboratory of Special Functional and Smart Polymer Materials of Ministry of Industry and Information Technology, School of Chemistry and Chemical Engineering, Northwestern Polytechnical University, Xi'an 710072, China.

[2]Department of Chemistry, Southern University of Science and Technology, Shenzhen 518055, China.

[3]State Key Laboratory of Cancer Biology, Department of Medical Genetics and Developmental Biology, Fourth Military Medical University, Xi'an 710032, China.

[4]Key Laboratory of Carbon Materials of Zhejiang Province, Wenzhou University, Wenzhou 325035, China.

Email: yongquan@nwpu.edu.cn, zhimintian@nwpu.edu.cn

Supplementary Figures

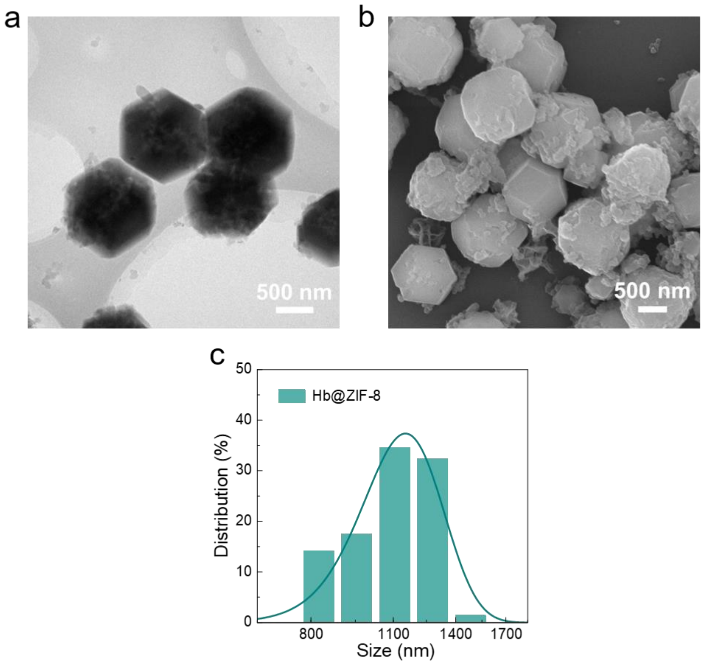

Supplementary Figure 1: a TEM image and **b** SEM image of Hb@ZIF-8. Three times each experiment was repeated independently with similar results. Representative images are shown. **c** Size distribution of the Hb@ZIF-8 based on 100 individual particles.

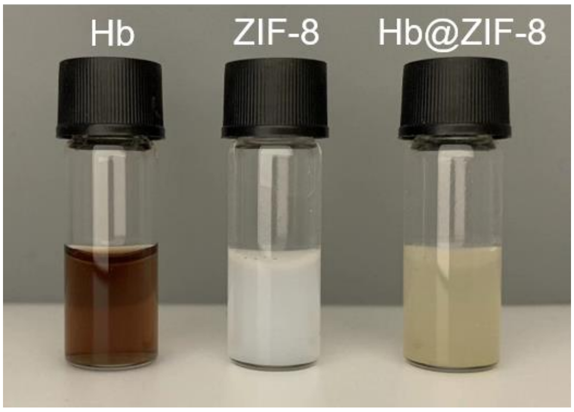

Supplementary Figure 2: Photographs of Hb, ZIF-8 and Hb@ZIF-8.

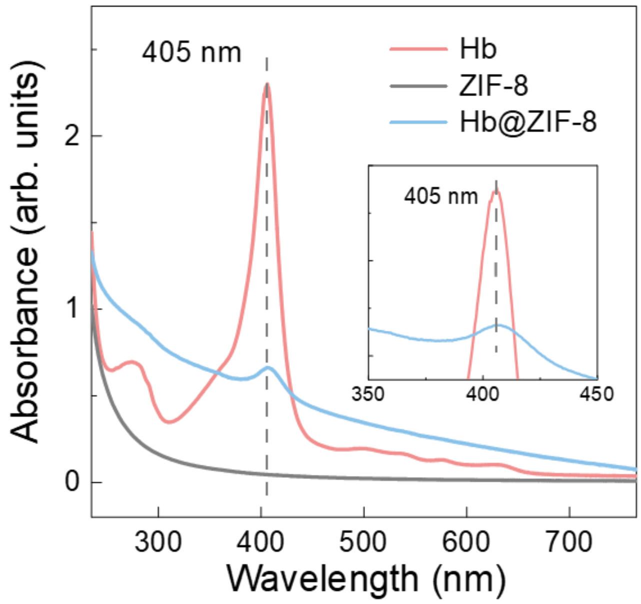

Supplementary Figure 3: UV-vis spectra of Hb, ZIF-8 and Hb@ZIF-8.

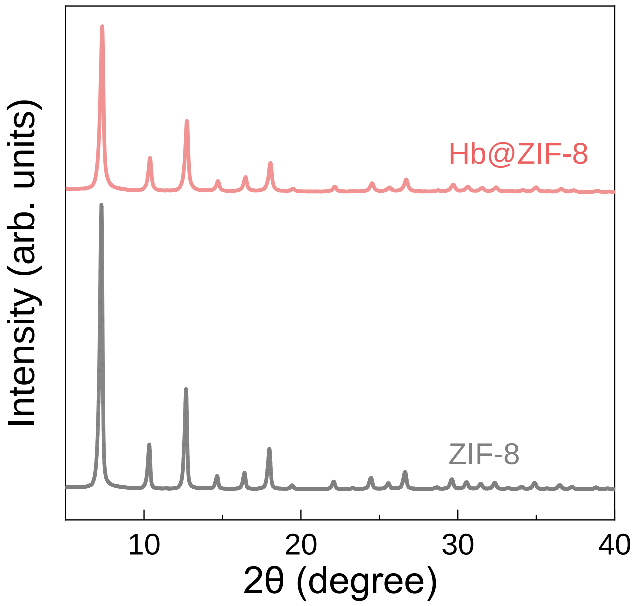

Supplementary Figure 4: XRD patterns of Hb@ZIF-8 and ZIF-8.

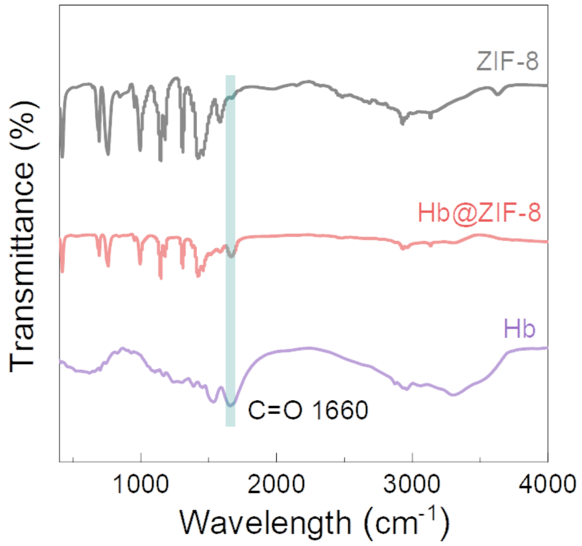

Supplementary Figure 5: FT-IR spectra of ZIF-8, Hb@ZIF-8 and Hb.

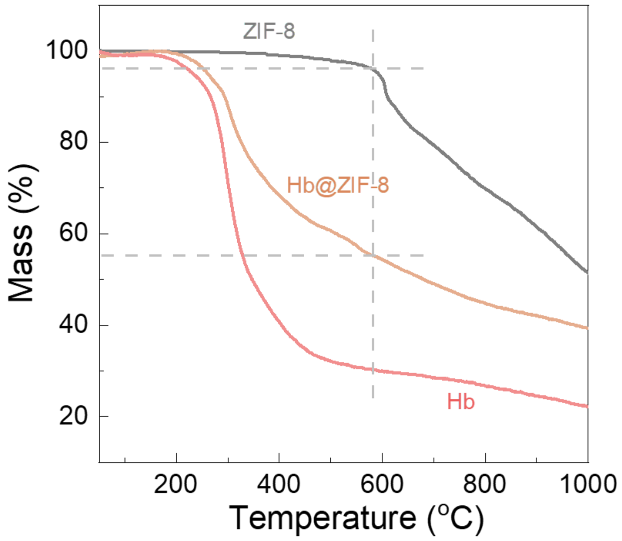

Supplementary Figure 6: TGA curves of ZIF-8, Hb@ZIF-8 and Hb under Ar atmosphere.

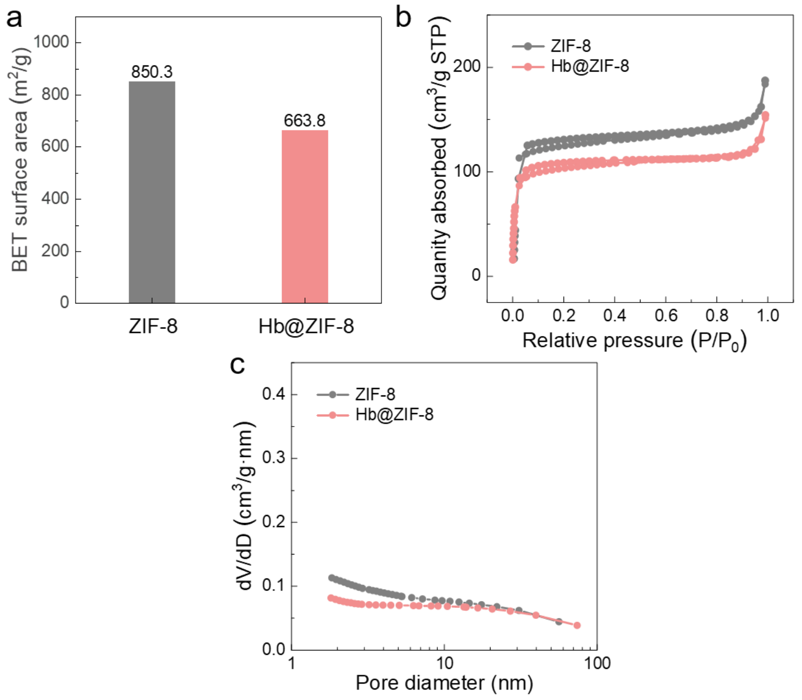

Supplementary Figure 7: a BET surface areas, **b** N_2 adsorption/desorption isotherms and **c** pore size distribution curves of ZIF-8 and Hb@ZIF-8.

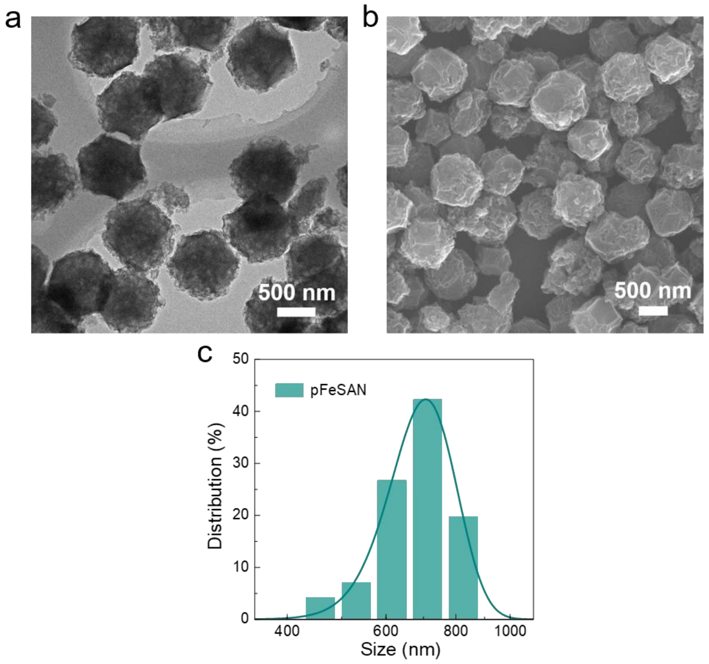

Supplementary Figure 8: a TEM image and **b** SEM image of pFeSAN. Three times each experiment was repeated independently with similar results. Representative images are shown. **c** Size distribution of the pFeSAN based on 100 individual particles.

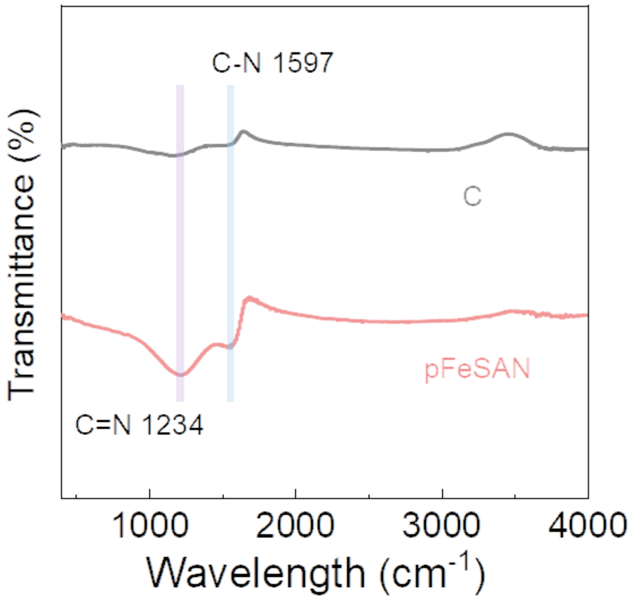

Supplementary Figure 9: FT-IR spectra of pFeSAN and C.

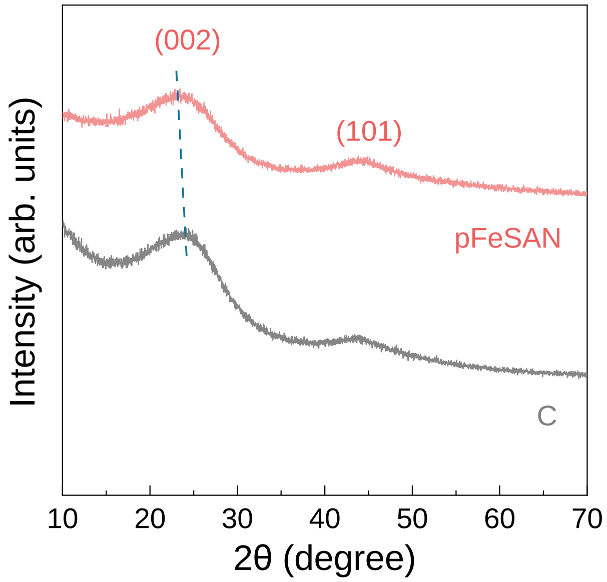

Supplementary Figure 10: XRD patterns of pFeSAN and carbon supports (C).

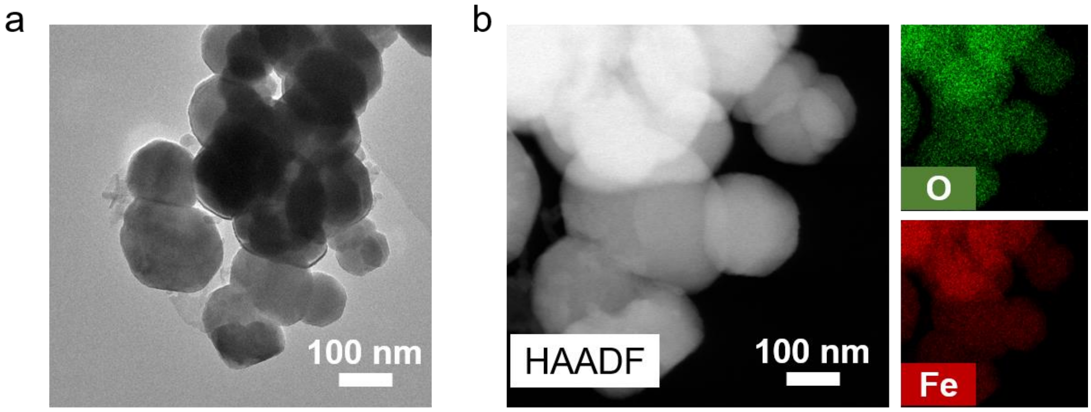

Supplementary Figure 11: **a** TEM image of Fe_3O_4. **b** HAADF-STEM image and the corresponding elemental mapping images of Fe_3O_4 showing the homogeneous distributions of Fe and O species. Three times each experiment was repeated independently with similar results. Representative images are shown.

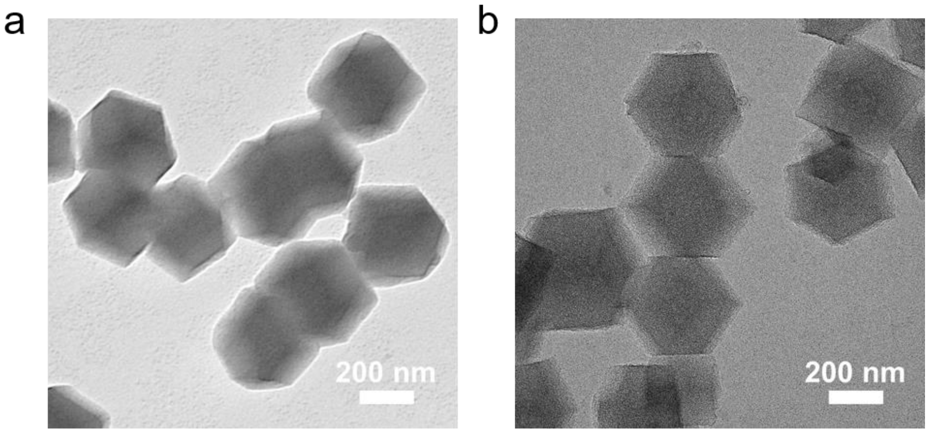

Supplementary Figure 12: TEM images of **a** ZIF-8 and **b** carbon supports (C). Three times each experiment was repeated independently with similar results. Representative images are shown.

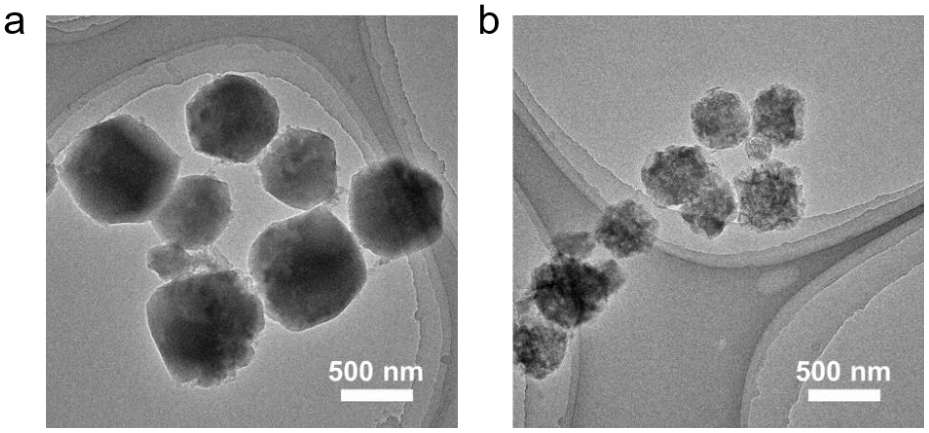

Supplementary Figure 13: TEM images of **a** BSA@ZIF-8 and **b** porous carbon supports (pC). Three times each experiment was repeated independently with similar results. Representative images are shown.

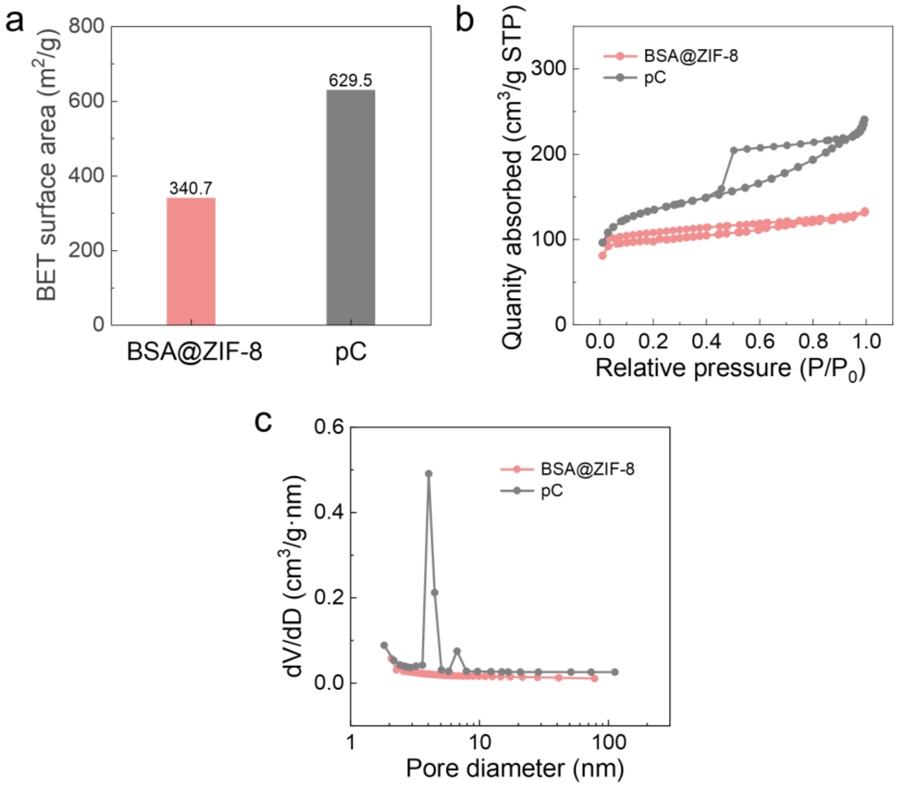

Supplementary Figure 14: **a** BET surface areas, **b** N_2 adsorption/desorption isotherms and **c** pore size distribution curves of BSA@ZIF-8 and porous carbon supports (pC).

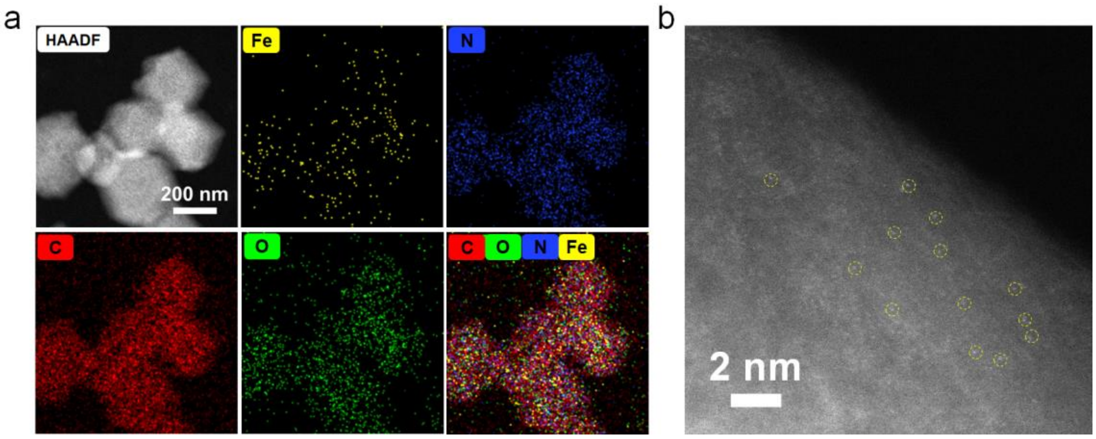

Supplementary Figure 15: a HAADF-STEM image and elemental mappings of Fe, C, N and O elements of FeSAN. **b** HAADF-STEM image of FeSAN showing the atomically dispersed Fe single atom sites as bright dots (yellow cycles marked single atoms). Three times each experiment was repeated independently with similar results. Representative images are shown.

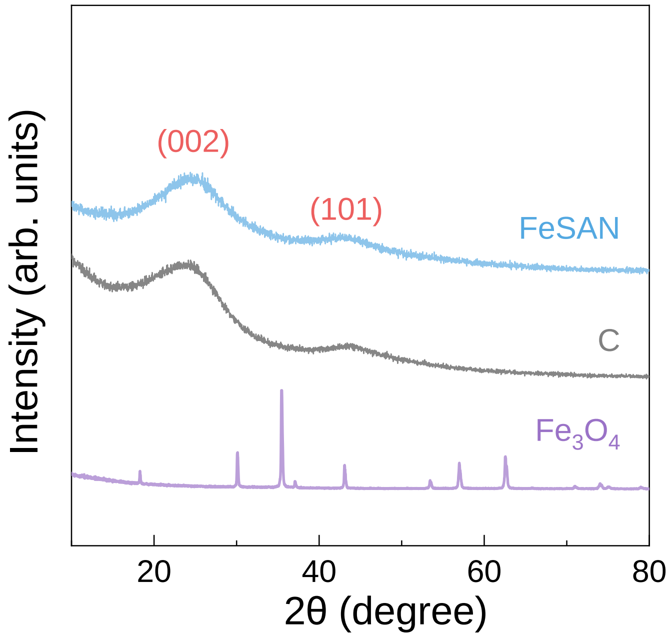

Supplementary Figure 16: XRD patterns of FeSAN, carbon supports (C) and Fe_3O_4.

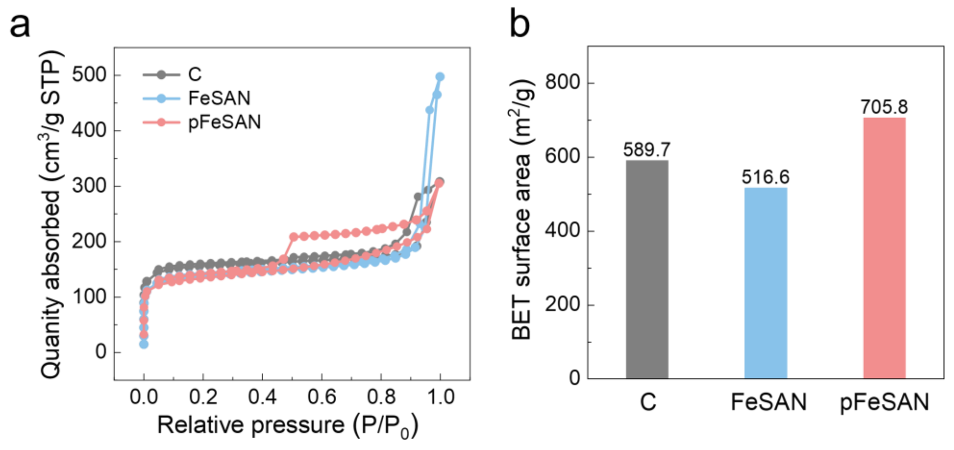

Supplementary Figure 17: a N_2 adsorption/desorption isotherms and **b** BET surface areas of carbon supports (C), FeSAN and pFeSAN.

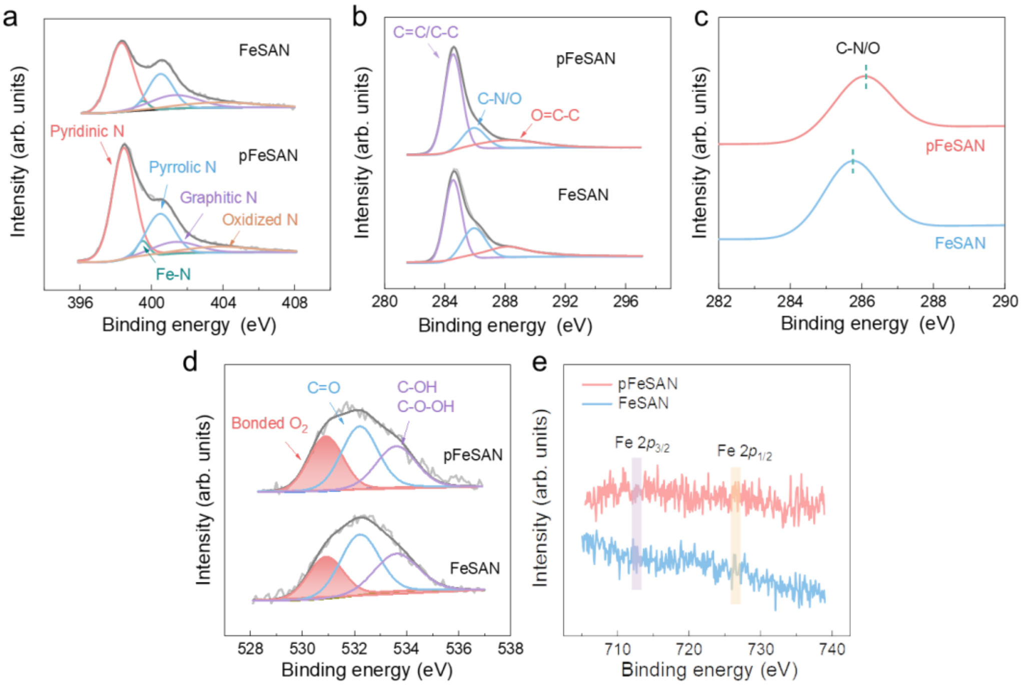

Supplementary Figure 18: XPS spectra of **a** N 1*s*, **b, c** C 1*s* and **d** O 1*s* for FeSAN and pFeSAN. **e** XPS spectra of Fe 2*p* for FeSAN and pFeSAN.

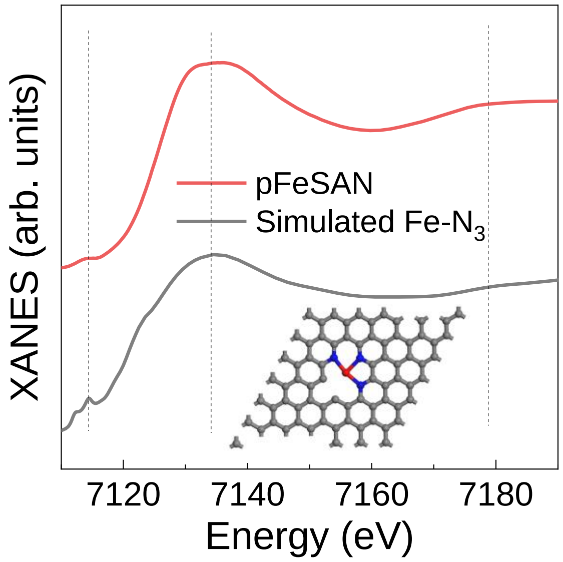

Supplementary Figure 19: Comparison of the experimental XANES curve (pFeSAN) with the calculated XANES data of Fe-N_3. The insets show the optimized Fe-N_3 structure. The relative pre-edge and white line peak of the simulated spectrum are consistent with the experiment.

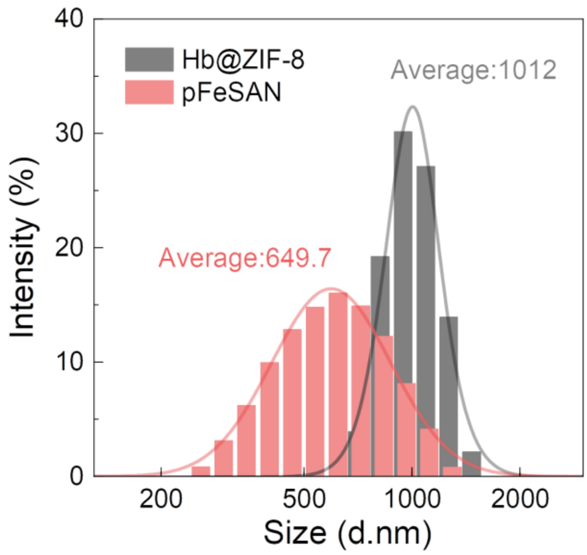

Supplementary Figure 20: The hydrodynamic diameters of Hb@ZIF-8 and pFeSAN dispersed in water tested by DLS.

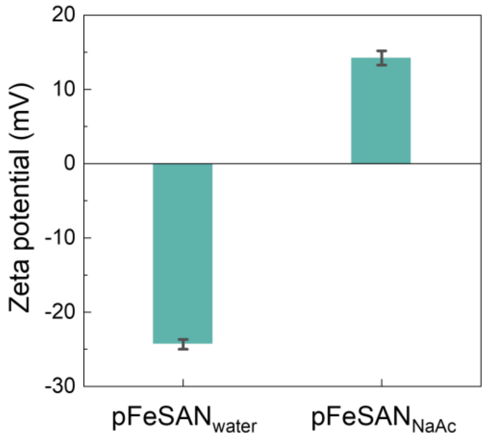

Supplementary Figure 21: Zeta potentials of pFeSAN in water or NaAc (pH 4.0). These data are presented as mean values ± SD (n = 3 independent experiments).

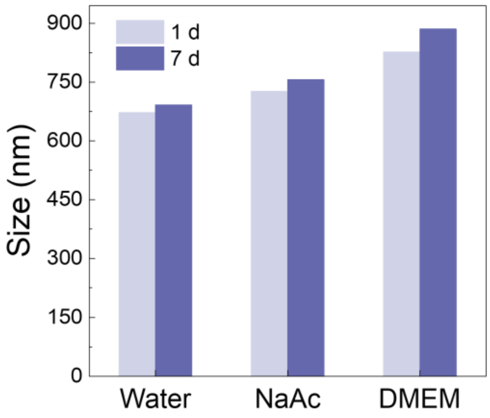

Supplementary Figure 22: The average hydrodynamic diameters of pFeSAN during the one-week co-incubation in various media.

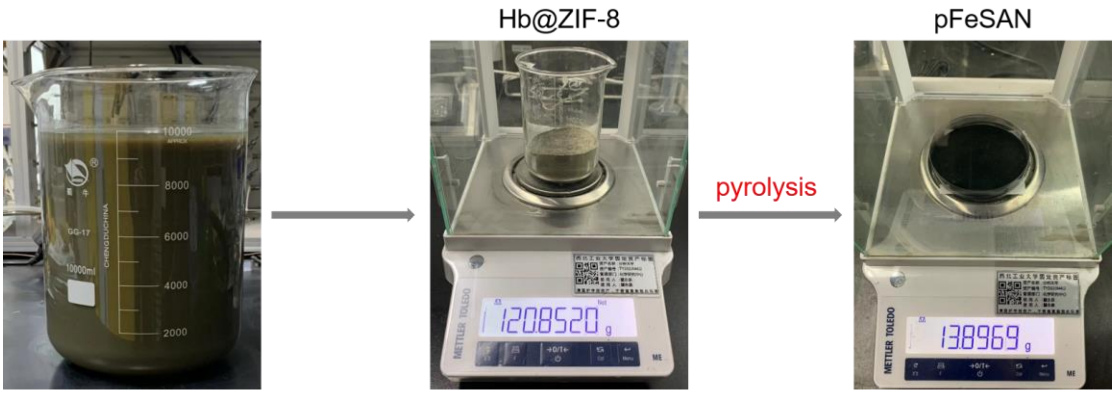

Supplementary Figure 23: Photographs of the reaction mixture, Hb@ZIF and pFeSAN.

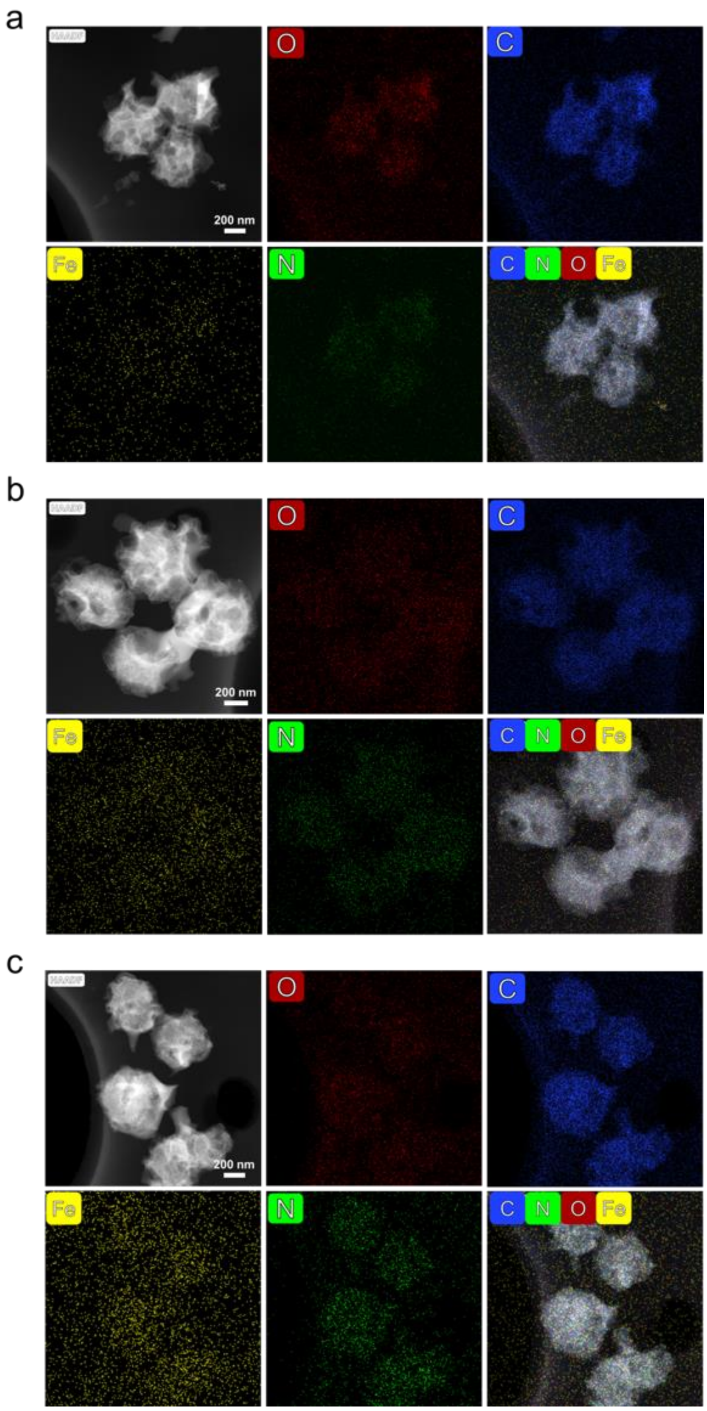

Supplementary Figure 24: HAADF-STEM images and corresponding EDS mapping of C, N, Fe and O in **a** pFeSAN-700, **b** pFeSAN-800 and **c** pFeSAN-1000. Three times each experiment was repeated independently with similar results. Representative images are shown.

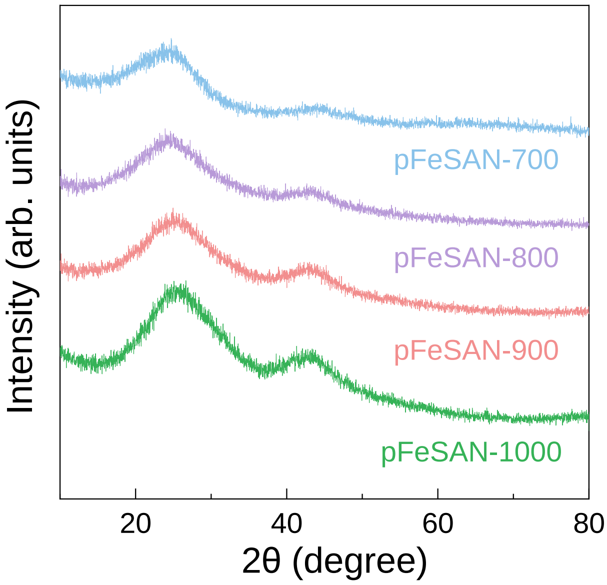

Supplementary Figure 25: XRD pattern of pFeSAN-700, pFeSAN-800, pFeSAN-900 and pFeSAN-1000.

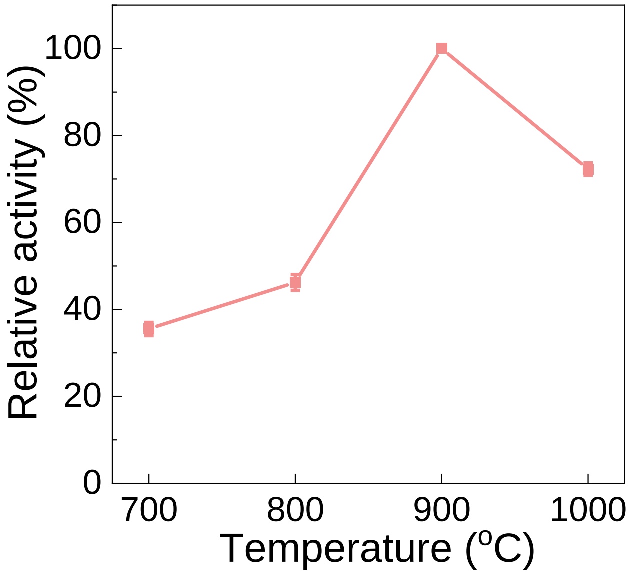

Supplementary Figure 26: The relative oxidase-like activity of pFeSAN prepared at various pyrolysis temperatures.

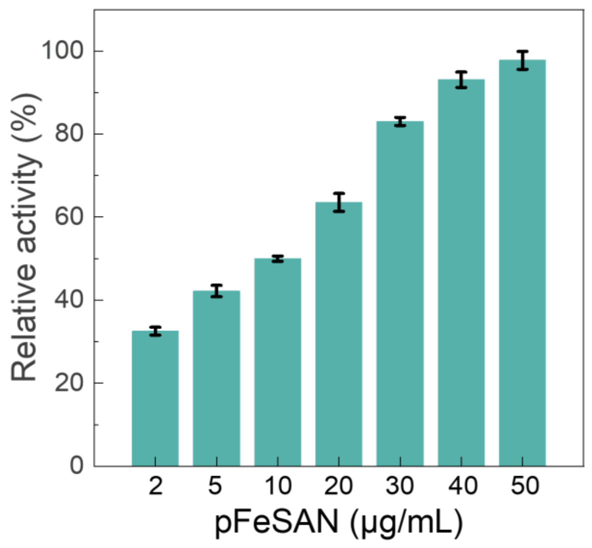

Supplementary Figure 27: Relative oxidase-like activity of pFeSAN prepared at various reaction concentrations. These data are presented as mean values ± SD (n = 3 independent experiments).

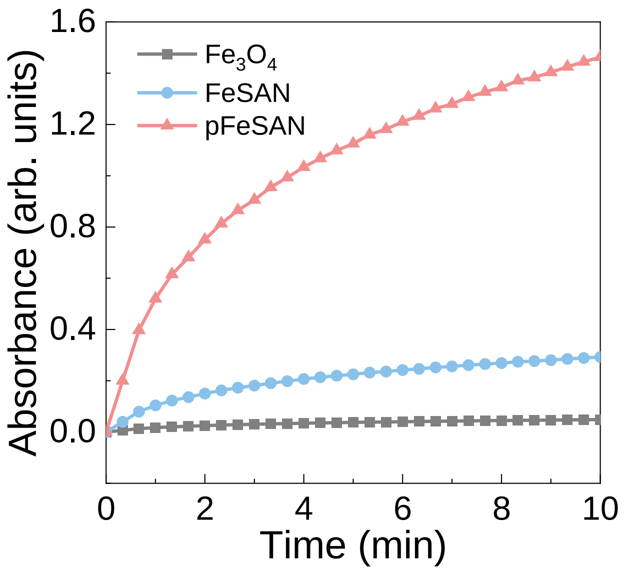

Supplementary Figure 28: Reaction-time curves of the TMB colorimetric reaction catalyzed by pFeSAN, FeSAN and Fe_3O_4.

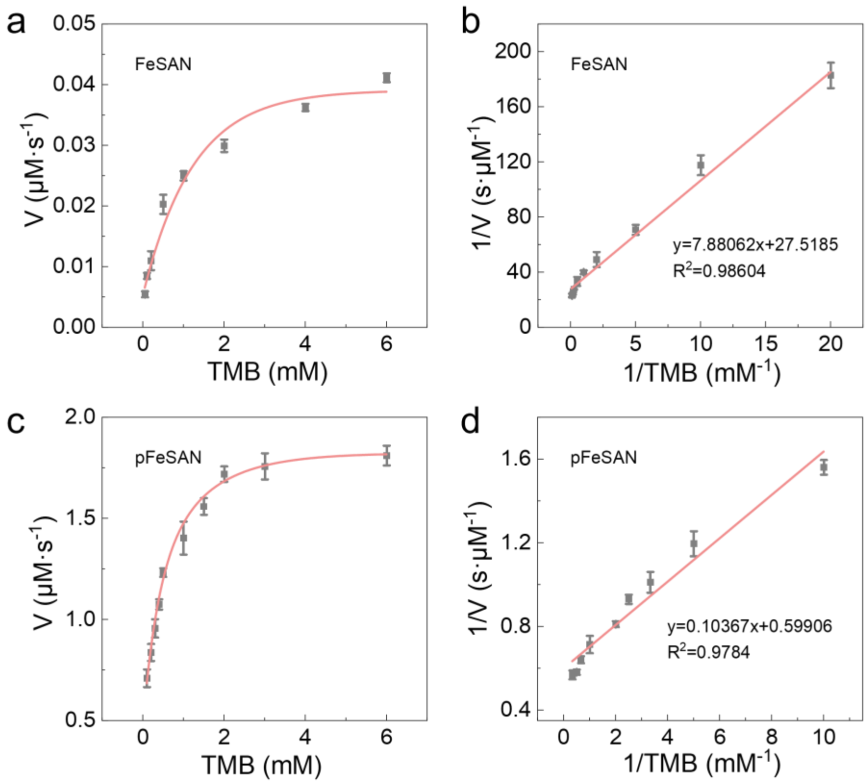

Supplementary Figure 29: Steady-state kinetic assays of FeSAN and pFeSAN as nanozyme for the oxidation of TMB. **a** Velocity of the reaction measured by FeSAN (20 μg/mL) towards various concentration of TMB. **b** Double-reciprocal plots of activity of FeSAN. **c** Velocity of the reaction measured by pFeSAN (20 μg/mL) towards various concentration of TMB. **d** Double-reciprocal plots of activity of pFeSAN. These data are presented as mean values ± SD (n = 3 independent experiments).

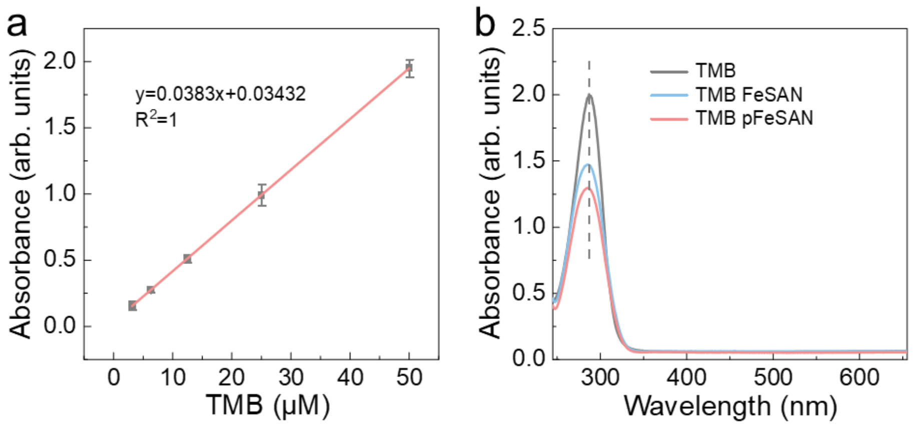

Supplementary Figure 30: a Linear curve of UV−vis absorbance of various concentrations of TMB solution (pH 7.0). These data are presented as mean values ± SD (n = 3 independent experiments). **b** UV-vis spectra of TMB (pH 7.0) after adsorption by FeSAN and pFeSAN at 25°C.

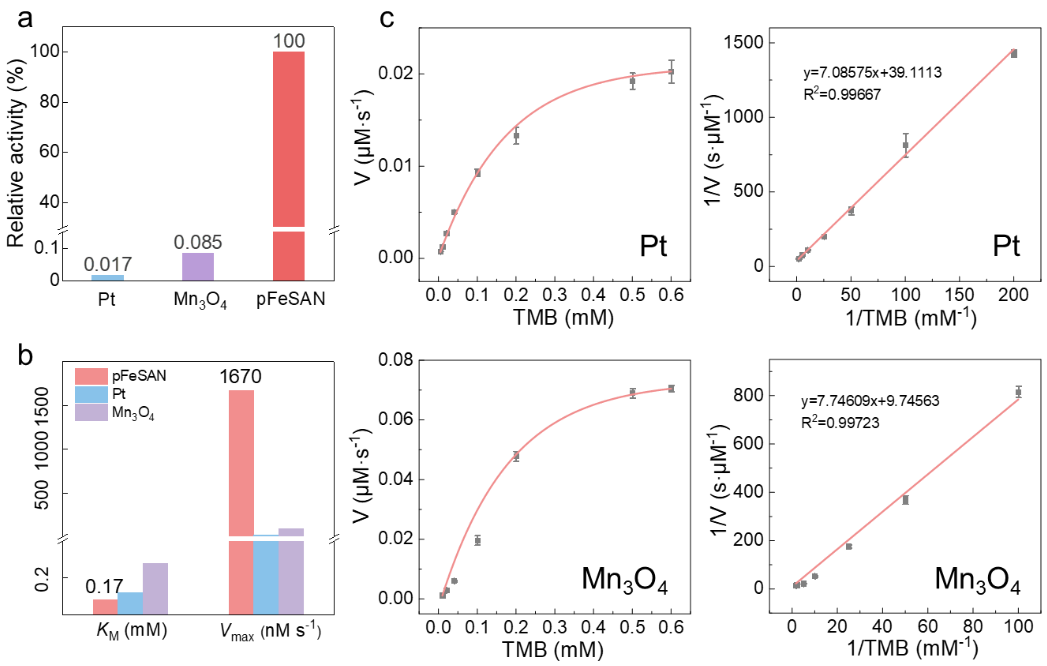

Supplementary Figure 31: Oxidase-like activity evaluation. **a**. Comparison of relative oxidase-like catalytic activities of different nanozymes. **b**. Comparison of kinetics for pFeSAN, Pt and Mn_3O_4. K_M is the Michaelis-Menten constant. V_{max} is the maximal reaction velocity. **c**. Steady-state kinetic assay of Pt and Mn_3O_4 with TMB as substrate. These data are presented as mean values ± SD (n = 3 independent experiments).

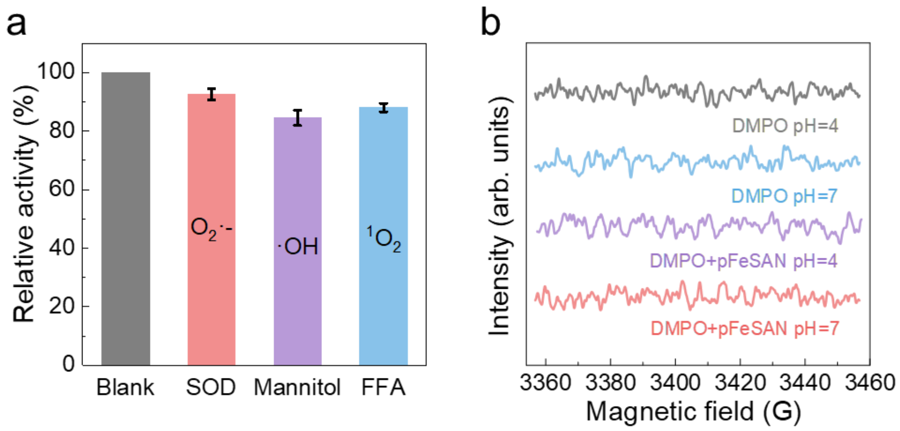

Supplementary Figure 32: a Effects of ROS scavengers (SOD, Mannitol and FFA) on the oxidase-like activity of pFeSAN. These data are presented as mean values ± SD (n = 3 independent experiments). **b** EPR spectra of pFeSAN + DMPO under different pH conditions.

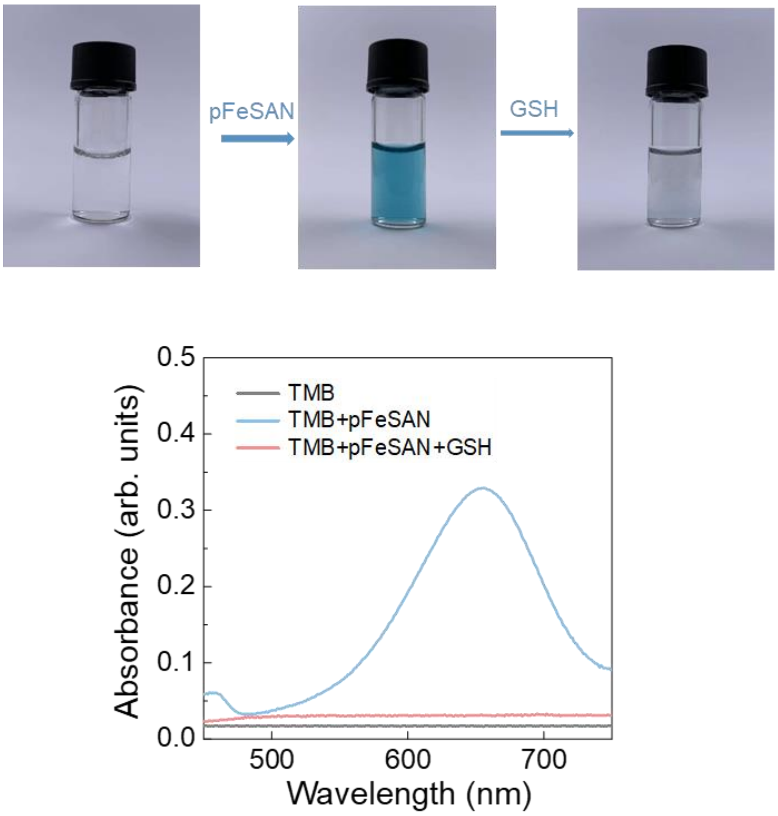

Supplementary Figure 33: Photographs and UV−vis absorption spectra of TMB, pFeSAN + TMB, pFeSAN + TMB + GSH.

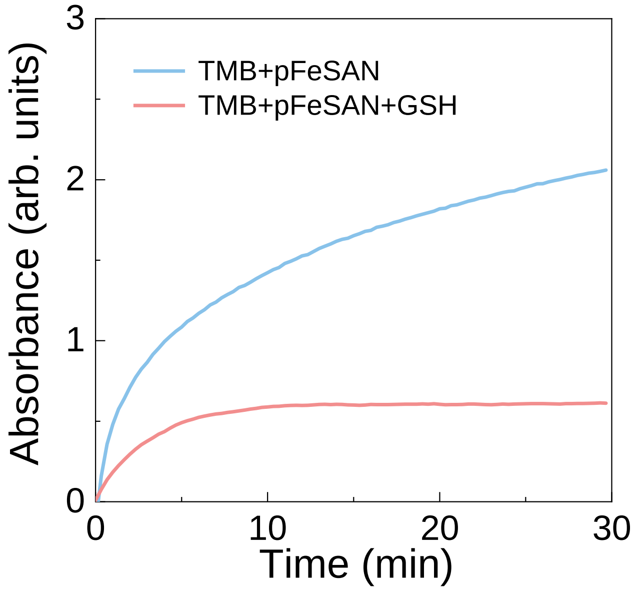

Supplementary Figure 34: Time-absorbance curves of absorbance changes at 652 nm in the presence of TMB + pFeSAN and TMB + pFeSAN + GSH for 30 min.

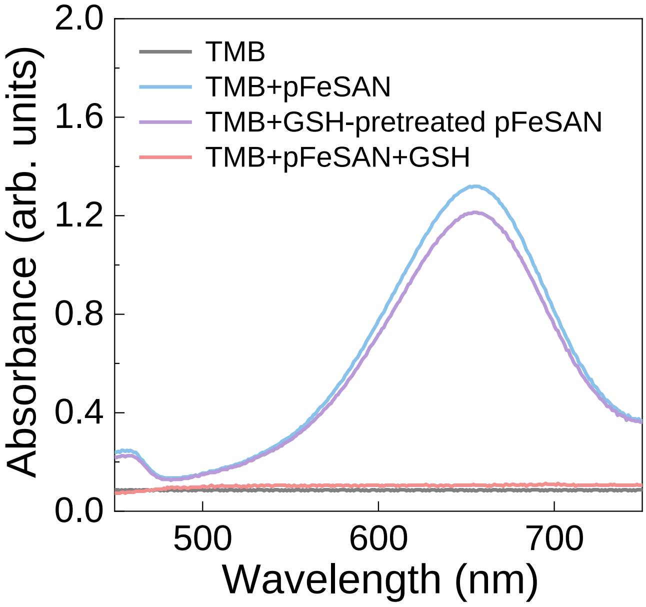

Supplementary Figure 35: UV−vis absorption spectra of TMB, pFeSAN + TMB, GSH-pretreated pFeSAN + TMB and pFeSAN + TMB + GSH.

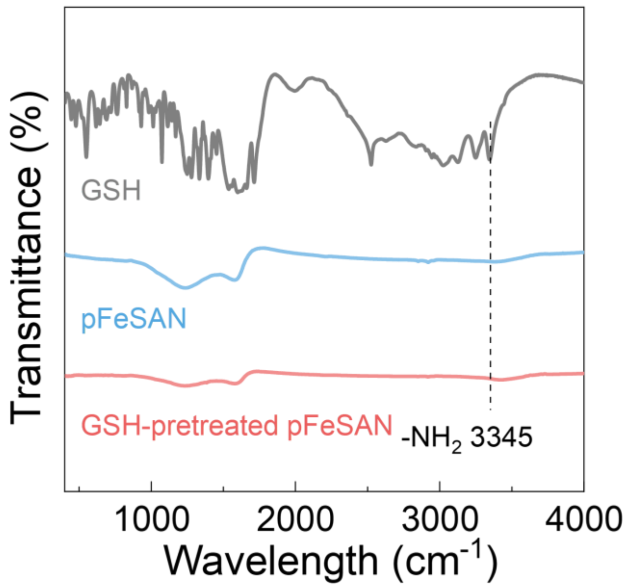

Supplementary Figure 36: FT-IR spectra of GSH, pFeSAN and GSH-pretreated pFeSAN.

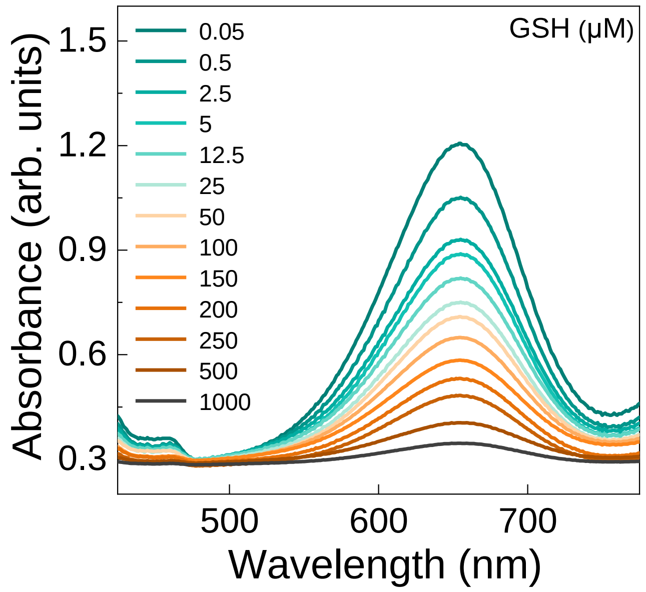

Supplementary Figure 37: UV-vis absorption spectra of TMB oxidized by pFeSAN in the presence of GSH with various concentrations (from top to bottom, 0.05, 0.5, 2.5, 5, 12.5, 25, 50, 100, 150, 200, 250, 500 and 1000 μM).

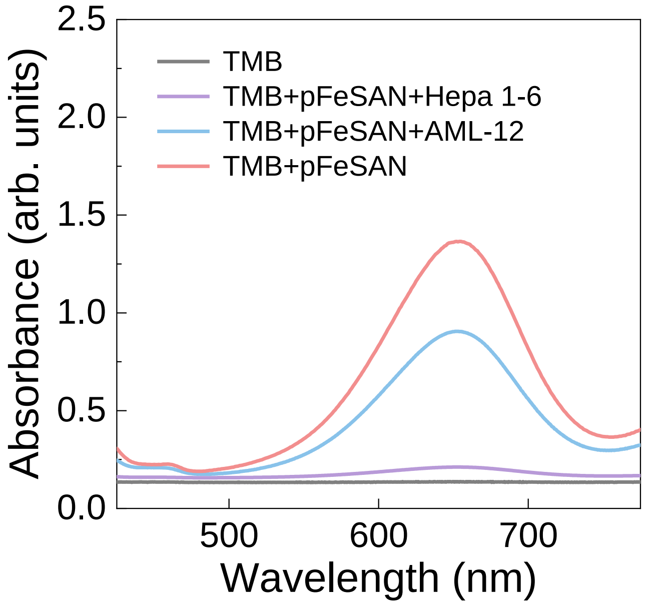

Supplementary Figure 38: UV-vis absorption spectra of TMB, TMB + pFeSAN + Hepa 1-6, TMB + pFeSAN + AML-12 and TMB + pFeSAN (Number of cells: 800000).

Supplementary Tables

Supplementary Table 1: EXAFS fitting parameters at the Fe K-edge various samples. [a]CN: coordination numbers, [b]R: bond distance, [c]σ^2: Debye-Waller factors, [d]ΔE_0: the inner potential correction, [e]R factor: goodness of fit.

Sample	Shell	CN[a]	R (Å) [b]	σ^2 (Å^2) [c]	ΔE_0 (eV) [d]	R factor[e]
Fe foil	Fe-Fe	8	2.47±0.01	0.0051	7.2±1.0	0.0034
	Fe-Fe	6	2.85±0.01	0.0065		
pFeSAN	Fe-N	2.8±0.3	1.91±0.02	0.0018	-2.4±0.8	0.0074

Supplementary Table 2: Comparison of kinetics for pFeSAN and FeSAN. These data are presented as mean values ± SD (n = 3 independent experiments).

Catalyst	K_M (mM)	V_{max} (μM s^{-1})	k_{cat} (s^{-1})	k_{cat}/K_M (mM^{-1}s^{-1})
pFeSAN	0.17	1.67	2.6×10^6	1.53×10^7
$Fe\text{-}N_4$	0.29	0.036	9.6×10^4	3.31×10^5

Supplementary Table 3: Comparison of the present methodology with other fluorescent and mimic enzyme-based methods for GSH detection.

Materials	Methods	Linear range	LOD	Reference
AuNPs	Colorimetry	1-40 µM	0.013 µM	1
Au nanoclusters	Colorimetry	2-25 µM	0.42 µM	2
PSMOF	Colorimetry	1-20 µM	0.68 µM	3
Acr^+-Mes	Colorimetry	0.1-40 µM	0.1 µM	4
MnO_2	Colorimetry	0.3-15 µM	0.11 µM	5
TiO_2/MoS_2	Colorimetry	0.05-1 µM	0.05 µM	6
hemicyanine	Fluorescent	1.0-15.0 mM	24.16 µM	7
MNPG	Fluorescence	0.2-20 µM	0.05 µM	8
TCDs	Fluorescent	0.2-175 µM	0.11 µM	9
Au nanoflowers	SERS	1.1-23.5 µM	0.4 µM	10
Cu-hemin-MOF	Colorimetry	0.05-2.5 µM	0.026 µM	11
$H_2TCPP–TiO_2–MMT$	Colorimetry	0.1-20 µM	0.057 µM	12
Fe-N-C SANs	Colorimetry	100-400 µM	78.3 µM	13
pFeSAN	Colorimetry	0.05-1000 µM	0.0024 µM	This work

Supplementary Table 4: Comparison between the present methodology and commercial kit for GSH detection.

Sample	GSH			
	Added (μM)	Found (μM)	Recovery (%)	RSD (%)
GSH assay kit	20	17	85	3.7
	40	43	107.5	2.5
	80	74.4	93	2.2
Our method	20	19.6	98	1.4
	40	41	102.5	2.2
	80	84.9	106.1	2.0

Supplementary References

1. Kumar, V. et al. Size-dependent synthesis of gold nanoparticles and their peroxidase-like activity for the colorimetric detection of glutathione from human blood serum. *ACS Sustainable Chem. Eng*. **6**, 7662–7675 (2018).
2. Feng, J. Y., Huang, P. C., Shi, S. Z., Deng, K.-Y. & Wu, F.-Y. Colorimetric detection of glutathione in cells based on peroxidase-like activity of gold nanoclusters: a promising powerful tool for identifying cancer cells. *Anal. Chim. Acta* **967**, 64–69 (2017).
3. Liu, Y. F. et al. Light-responsive metal organic framework as an oxidase mimic for cellular glutathione detection. *Anal. Chem*. **91**, 8170–8175 (2019).
4. Du, J. Y., Wang, J. H., Huang, W., Deng, Y. Q. & He, Y. Visible light-activatable oxidase mimic of 9-mesityl-10-methylacridinium ion for colorimetric detection of biothiols and logic operations. *Anal. Chem*. **90**, 9959–9965 (2018).
5. Zhang, X. D., Mao, X. X., Li, S. Q., Dong, W. F. & Huang, Y. M. Tuning the oxidase mimics activity of manganese oxides via control of their growth conditions for highly sensitive detection of glutathione. *Sens. Actuators, B* **258**, 80–87 (2018).
6. Zhu, W. D., Chi, M. Q., Gao, M., Wang, C. & Lu, X. F. Controlled synthesis of titanium dioxide/molybdenum disulfide core-shell hybrid nanofibers with enhanced peroxidase-like activity for colorimetric detection of glutathione. *J. Colloid Interface Sci*. **528**, 410–418 (2018).
7. Qi, S. J. et al. A colorimetric and ratiometric fluorescent probe for highly selective detection of glutathione in the mitochondria of living cells. *Sens. Actuators, B* **270**, 459–465 (2018).
8. Zhang, H. J., Chen, J., Yang, Y. L., Wang, L., Li, Z. & Qiu, H. D. Discriminative detection of glutathione in cell lysates based on oxidase-like activity of magnetic nanoporous graphene. *Anal. Chem*. **91**, 5004–5010 (2019).
9. Yang, R., Guo, X. F., Jia, L. H. & Zhang, Y. A fluorescent “on-off-on” assay for selective recognition of Cu(II) and glutathione based on modified carbon

nanodots, and its application to cellular imaging. *Microchim. Acta* **184**, 1143–1150 (2017).

10. Wang, W. K., Zhang, L. M., Li, L. & Tian, Y. A single nanoprobe for ratiometric imaging and biosensing of hypochlorite and glutathione in live cells using surface-enhanced raman scattering. *Anal. Chem*. **88**, 9518–9523 (2016).
11. Chen, X. L. et al. Colorimetric and fluorescent dual-identification of glutathione based on its inhibition on the 3D ball-flower shaped Cu-hemin-MOF's peroxidase-like activity. *Microchim Acta* **187**, 601 (2020).
12. Zhu, X. X. et al. Novel "on-off" colorimetric sensor for glutathione based on peroxidase activity of montmorillonite-loaded TiO_2 functionalized by porphyrin precisely controlled by visible light. *ACS Sustainable Chem. Eng*. **7**, 18105–18113 (2019).
13. Lu, W., Chen, S. H., Zhang, H. F., Qiu, J. J. & Liu, X. Y., Fe-N-C single atom nanozymes with dual enzyme-mimicking activities for colorimetric detection of hydrogen peroxide and glutathione. *J. Materiomics* **8**, 1251–1259 (2022).
